# Clinical Course in Chronic Subdural Hematoma Patients Aged 18–49 Compared to Patients 50 Years and Above: A Multicenter Study and Meta-Analysis

**DOI:** 10.3389/fneur.2019.00311

**Published:** 2019-04-05

**Authors:** Jiri Bartek, Kristin Sjåvik, Sanjay Dhawan, Lisa M. Sagberg, Helena Kristiansson, Fredrik Ståhl, Petter Förander, Clark C. Chen, Asgeir S. Jakola

**Affiliations:** ^1^Department of Neurosurgery, Karolinska University Hospital, Stockholm, Sweden; ^2^Department of Clinical Neuroscience and Department of Medicine, Karolinska Institutet, Stockholm, Sweden; ^3^Department of Neurosurgery, Copenhagen University Hospital Rigshospitalet, Copenhagen, Denmark; ^4^Department of Neurosurgery, University Hospital of North Norway, Tromsø, Norway; ^5^Department of Neurosurgery, University of Minnesota, Minneapolis, MN, United States; ^6^Department of Neurosurgery, St. Olavs University Hospital, Trondheim, Norway; ^7^Department of Public Health and Nursing, Norwegian University of Science and Technology, Trondheim, Norway; ^8^Department of Neuroradiology, Karolinska University Hospital, Stockholm, Sweden; ^9^Department of Neurosurgery, Sahlgrenska University Hospital, Gothenburg, Sweden; ^10^Institute of Neuroscience and Physiology, University of Gothenburg, Sahlgrenska Academy, Gothenburg, Sweden

**Keywords:** chronic subdural hematoma, young, neurosurgery, symptoms, clinical course, morbidity, mortality, meta-analysis

## Abstract

**Objective:** Chronic Subdural Hematoma (cSDH) is primarily a disease of elderly, and is rare in patients <50 years, and this may in part be related to the increased brain atrophy from 50 years of age. This fact may also influence clinical presentation and outcome. The aim of this study was to study the clinical course with emphasis on clinical presentation of cSDH patients in the young (<50 years).

**Methods:** A retrospective review of a population-based cohort of 1,252 patients operated for cSDH from three Scandinavian neurosurgical centers was conducted. The primary end-point was difference in clinical presentation between the patients <50 y/o and the remaining patients (≥50 y/o group). The secondary end-points were differences in perioperative morbidity, recurrence and mortality between the two groups. In addition, a meta-analysis was performed comparing clinical patterns of cSDH in the two age groups.

**Results:** Fifty-two patients (4.2%) were younger than 50 years. Younger patients were more likely to present with headache (86.5% vs. 37.9%, *p* < 0.001) and vomiting (25% vs. 5.2%, *p* < 0.001) than the patients ≥50 y/o, while the ≥50 y/o group more often presented with limb weakness (17.3% vs. 44.8%, *p* < 0.001), speech impairment (5.8% vs. 26.2%, *p* = 0.001) and gait disturbance or falls (23.1% vs. 50.7%, *p* < 0.001). There was no difference between the two groups in recurrence, overall complication rate and mortality within 90 days. Our meta-analysis confirmed that younger patients are more likely to present with headache (*p* = 0.015) while the hemispheric symptoms are more likely in patients ≥50 y/o (*p* < 0.001).

**Conclusion:** Younger patients with cSDH present more often with signs of increased intracranial pressure, while those ≥50 y/o more often present with hemispheric symptoms. No difference exists between the two groups in terms of recurrence, morbidity, and short-term mortality. Knowledge of variations in clinical presentation is important for correct and timely diagnosis in younger cSDH patients.

## Introduction

Chronic subdural hematoma (cSDH) is a diagnosis with increasing prevalence among the older. Nevertheless, cSDH is also seen in younger patients (previously defined as <50 years in literature), although less commonly (1–5). And while cSDH in the young is often associated with predisposing condition i.e., arachnoid cysts, coagulation disorders, or ventriculo-peritoneal shunts, even cSDH without predisposing condition has been reported, often in connection with head trauma (6, 7). Correct interpretation of clinical presentation is important for timely diagnosis. Nevertheless, the reports from literature on the clinical presentation of younger patients with cSDH are varied and often conflicting (1–5). Also, even though surgical evacuation is the main treatment modality in cSDH patients, little is known as to the recurrence rates, morbidity and mortality of young patients with surgically treated cSDH. A large-scale, population-based multicenter study is warranted to benchmark the clinical course and outcome of younger patients with cSDH.

In this population-based multicenter study of adult patients with cSDH, we assessed the clinical course and outcome in patients <50 years, and compared results to the group of patients ≥50 y/o.

## Materials and Methods

### Eligible Study Patients

Adult patients (≥18 years old) with a cSDH (defined as hypodensity, hyperdensity, and/or combined hypo- and hyper-density on computed tomography (CT) treated with burr hole evacuation between January 1, 2005 and December 31, 2010 at the departments of Neurosurgery at St. Olavs University Hospital (Trondheim, Norway), Karolinska University Hospital (Stockholm, Sweden) and the University Hospital of North Norway (Tromsø, Norway) were included. Patients operated intracranially within the past 6 months, patients with ventriculo-peritoneal shunts, with cSDH in relation to arachnoidal cysts were excluded. The Nordic health care system with regional referral practice to neurosurgical centers ensures a representative population-based study, with data gathering for this database done by experienced medical staff. As reported previously, there was no differences between regions in the age and sex adjusted incidence rates (*p* = 0.096, data not shown) (8).

### Treatment Characteristics

Diagnosis was ascertained by a computed tomography (CT) and/or magnetic resonance imaging (MRI) in all patients. Anticonvulsants were not routinely administered unless the patient has experienced convulsions. cSDH evacuation was performed according to local practice (8). At all centers taking part in the study, surgery was offered to all patients with symptomatic cSDH as first-line treatment regardless of age.

### Study and Outcome Variables

According to previously calculated (9) age-adjusted incidence rates for the different age groups the incidence is very low, 0.6/100.000 among patients 18–49 y/o, while this rise to 7.1/100.000 (50–66 y/o) and up to 59.5/100.000 (80–89 y/o). This information together with knowledge of brain atrophy increasing after 50 years of age (10), and that this age group of 18–49 years old was <5% of our population-based cohort argued for a “younger group” aged 18–49 years old.

Data collection for this manuscript was done by experienced medical staff using the electronic patient records at each center, with data quality confirmed by the principal investigator based upon quality control. End of follow-up was 01.01.2012, thus all patients were followed at least 1 year or until death. Recurrence was defined as same-sided chronic subdural hematoma within 6 months following index surgery. Patient characteristics included data on the pre-operative status including comorbidity and residential situation (home without assistance, home care and institution) (11), Radiology characteristics included hematoma location (right, left, and bilateral), density (hypodense, mixed, isodens, and hyperdens) and midline shift. Further, the radiology was reviewed to ensure that no apparent bleeding source (such as an AVM, fistula, tumor etc.) was present in any of the included patients, nor did any included patient have any structural abnormalities. Also, upon review, the younger group did not have any signs of early atrophy. Adverse events within 30 days were classified according to Landriel Ibanez et al. (12), with severe morbidity classified as Landriel grade III. Mortality was assessed at 1 and 3 months. Recurrent cSDH was defined as any same-sided cSDH treatment within 6 months of index surgery.

Overall recurrence and survival were illustrated by Kaplan-Meier analysis and compared with log-rank test. According to protocol, we evaluated recurrence up to 6 months after surgery as done by others (13), since most recurrences occur early and a longer time-span would increase chance of including *de novo* cases rather than treatment failures. We had 6 months follow-up on all patients, however in the analysis of overall survival patients were censored at end of follow up if still alive.

### Meta-Analysis

Systematic review of literature and meta-analysis was performed to determine if differences exist between <50 y/o and ≥50 y/o patients with subdural hematoma in terms of sex, medical history, presenting symptoms, imaging findings, and complication rates. The cut off age was chosen as 50 years for the meta-analysis except for 4 studies with 40 and 45 years as the cut off age (these were included due to their large cohort size).

**Figure 1:**
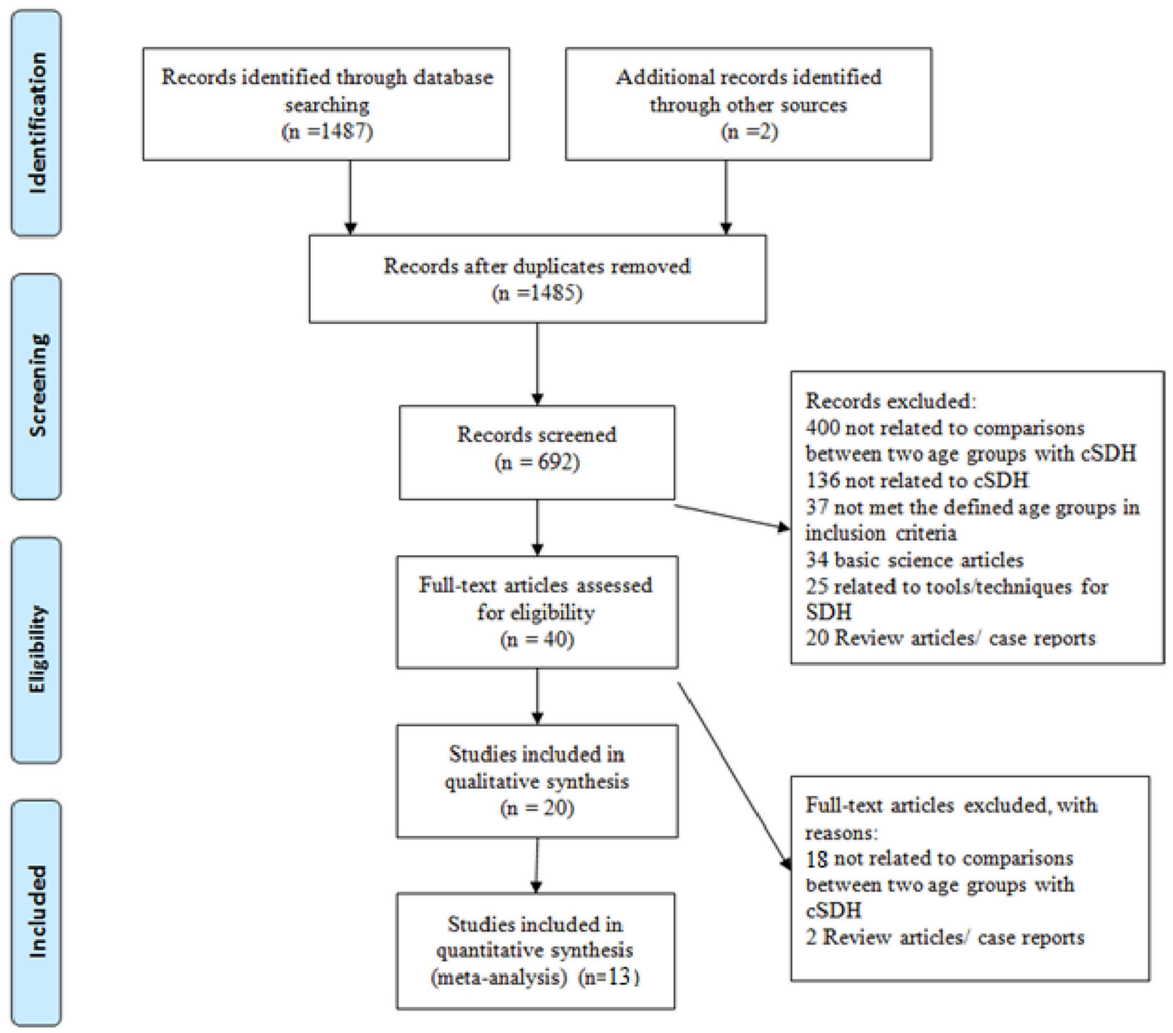
PRISMA Flow Diagram for search strategy and literature selection.

The meta-analysis was performed in accordance with the Preferred Reporting Items for Systematic Reviews and Meta-Analysis (PRISMA) guidelines (14). A comprehensive PubMed database search was conducted on May 17th, 2018 using the term (subdural hematoma OR SDH) [Title/Abstract] AND (young^*^ OR elderly^*^ OR adult^*^ or age groups^*^ OR comparison) [Title/Abstract] (Figure 1).

Articles were included if they: (1) were written in English (or an English translation was available), (2) involved human subjects, (3) were fully published peer-reviewed clinical articles, (4) had at least an abstract available, (5) included a comparison of the younger and older patients with cSDH. Case reports, articles related to basic sciences and other operative techniques in stereotaxy; editorials, commentaries, or conference proceedings were excluded. The literature search was not restricted by the date of publication of the articles. In the meta-analysis, hemispheric symptoms were defined as occurrence of sensory or motor deficit, gait disturbance, speech impairment, mental status change, or seizure. Bleeding tendency was considered to be present if the patient was on anti-platelets or anti-coagulation therapy, or with a hematologic disorder or liver disease.

### Statistical Analysis

SPSS version 24.0 (SPSS, Inc., Chicago, IL, USA) was used for analysis, while the meta-analysis was performed using Comprehensive Meta-Analysis software (Version 3.3070, BioStat, Englewood, NJ, USA). Significance was defined as *P* < 0.05, with no adjustments for multiple comparisons. Continuous variables were analyzed using an unpaired two-tailed *t*-test for normally distributed data. Continuous data with skewed distribution were analyzed with the Mann-Whitney U-test. Discrete variables were compared using a Chi-square analysis. The meta-analysis was done by pooling results using a random-effects model. Heterogeneity was categorized as low (25–50%), moderate (50–75%), or high (>75%) (15). Publication bias was evaluated using funnel plots, Egger's regression intercept test, and Duval and Tweedie's trim and fill test (16, 17).

## Results

One thousand two hundred and fifty two patients operated for cSDH were included. We identified 52 patients (4.2%) with primary operations for cSDH evacuation in younger patients aged 49 or less (<50 y/o group). The median age in the respective age groups was 40.9 years in the younger group and 75.4 years in the ≥50 y/o group. The median follow-up time (from surgery until end of follow-up) was 42 months in the younger group and 43 months in the ≥50 y/o group.

Younger patients presented more often with headache (86.5% vs. 37.9%, *p* < 0.001) and vomiting (25.0% vs. 5.2%, *p* < 0.001), while the patients ≥50 y/o more often presented with limb weakness (17.3% vs. 44.8%, *p* < 0.001), speech impairment (5.8% vs. 26.2%, *p* = 0.001) and gait disturbance or falls (23.1% vs. 50.7%, *p* < 0.001). No difference between groups was seen with respect to cognitive deterioration, acute confusion, drowsiness or coma, seizures, incontinence and/or visual disturbances. See Table 1 for further details.

**Table 1:** Clinical presentation in patients with cSDH <50 y/o vs. ≥50 y/o.

**Characteristics**	**18-49 y/o*n* = 52 (%)**	**≥50 y/o*n* = 1,200 (%)**	***p*-value**
Limb weakness	9 (17.3)	538 (44.8%)	<0.001
Headache	45 (86.5%)	455 (37.9%)	<0.001
Speech impairment	3 (5.8)	314 (26.2%)	0.001
Gait disturbance or falls	12 (23.1)	608 (50.7)	<0.001
Cognitive deterioration	8 (15.4)	283 (23.6)	0.17
Acute confusion	6 (11.5)	251 (20.9)	0.10
Drowsiness or coma	6 (11.5)	197 (16.4)	0.35
Seizure	1 (1.9)	37 (3.1)	0.63
Incontinence	0	55 (4.6)	0.11
Visual disturbances	1 (1.9)	20 (1.7)	0.89
Vomiting	13 (25.0)	62 (5.2)	<0.001

Compared to the patients ≥50 y/o, the younger patients had less comorbidities (13.5% vs. 34.6%, *p* = 0.002), were more independent (*p* < 0.001) and used less anti-platelets (1.9% vs. 28.1%, *p* < 0.001) and anti-coagulants (5.8% vs. 17.9%, *p* = 0.02). Pre-operatively, younger patients had on average a smaller hematoma diameter (18 mm vs. 22 mm, *p* < 0.001), but midline shift was similar. Younger patients were less often operated with local anesthesia and sedation only (84.6% vs. 94.7%, *p* = 0.002). See Table 2 for further details.

**Table 2:** Baseline and per-operative characteristics in patients with cSDH <50 y/o vs. ≥50 y/o.

**Characteristics**	**18–49 y/o*n* = 52 (%)**	**≥50 y/o*n* = 1,200 (%)**	***p*-value**
Median age, years (SD)	40.9 (6.7)	75.4 (10.4)	<0.001
Mean follow-up in months (SD)	42 (21)	43 (19)	0.65
Sex male	37 (71.2)	835 (69.6)	0.81
CCI^*^ > 1	7 (13.5)	415 (34.6)	0.002
Bilateral surgery (missing, *n* = 3)	5 (9.6)	212 (18.2)	0.11
Hypodense or mixed density Missing 89	33 (66.0)	830 (74.6)	0.18
Largest diameter mean mm (SD)	18.0 (6.0)	22.0 (6.2)	<0.001
Midline shift, mean mm (SD)	8.5 (5.1)	7.7 (4.7)	0.24
GCS^**^ in categories			0.38
13–15	49 (94.2)	1061 (89.8)	
9–12	3 (5.8)	82 (6.9)	
3–8	0	39 (3.3)	
Missing 18			
Antiplatelet drugs Missing 2	1 (1.9)	337 (28.1)	<0.001
Anticoagulants Missing 2	3 (5.8)	215 (17.9)	0.02
Home w/o care	51 (98.1)	880 (73.3)	<0.001
Home with support	1 (1.9)	205 (17.1)	
Institution	0	110 (9.4)	
Surgical procedure			<0.001
Passive drain	13 (25.0)	323 (26.9)	
Continuous	5 (9.6)	141 (11.8)	
Subgaleal/subperiostal	30 (57.7)	729 (60.8)	
Burrhole without drainage	4 (7.7)	7 (0.6)	
Local anesthesia + sedation Missing 35	44 (84.6)	1,133 (94.7)	0.002

* *Charlson Comorbidity Index*.

** *Glasgow Coma Scale*.

### Outcome

Younger patients had more often a clinical (*p* = 0.002) and/or radiological (*p* = 0.005) follow-up after surgery. No difference between groups was seen with respect to recurrence (Figure 4) and complications (Table 3). There was a difference in overall survival between groups (*p* = 0.002, Figure 5). See also Table 3 for further details.

**Table 3:** Outcome in patients with cSDH <50 y/o vs. ≥50 y/o.

**Characteristics**	**18–49 y/o*n* = 52 (%)**	**≥50 y/o*n* = 1,200 (%)**	***p*-value**
CT control planned Missing 41	26 (50.0)	364 (31.4)	0.005
Clinical control planned at hospital Missing 42	36 (69.2)	546 (47.2)	0.002
Recurrence	8 (15.4)	161 (13.4)	0.68
Overall complication	2 (3.8)	108 (9.0)	0.20
Complication Landriel grade ≥3	0	37 (3.1)	0.20
Mortality 30 days Missing 2	0	42 (3.5)	0.17
Mortality 90 days Missing 2	1 (1.9)	76 (6.3)	0.19

### Meta-Analysis Results With <50 Years as Cut-Off

**Figure 2:**
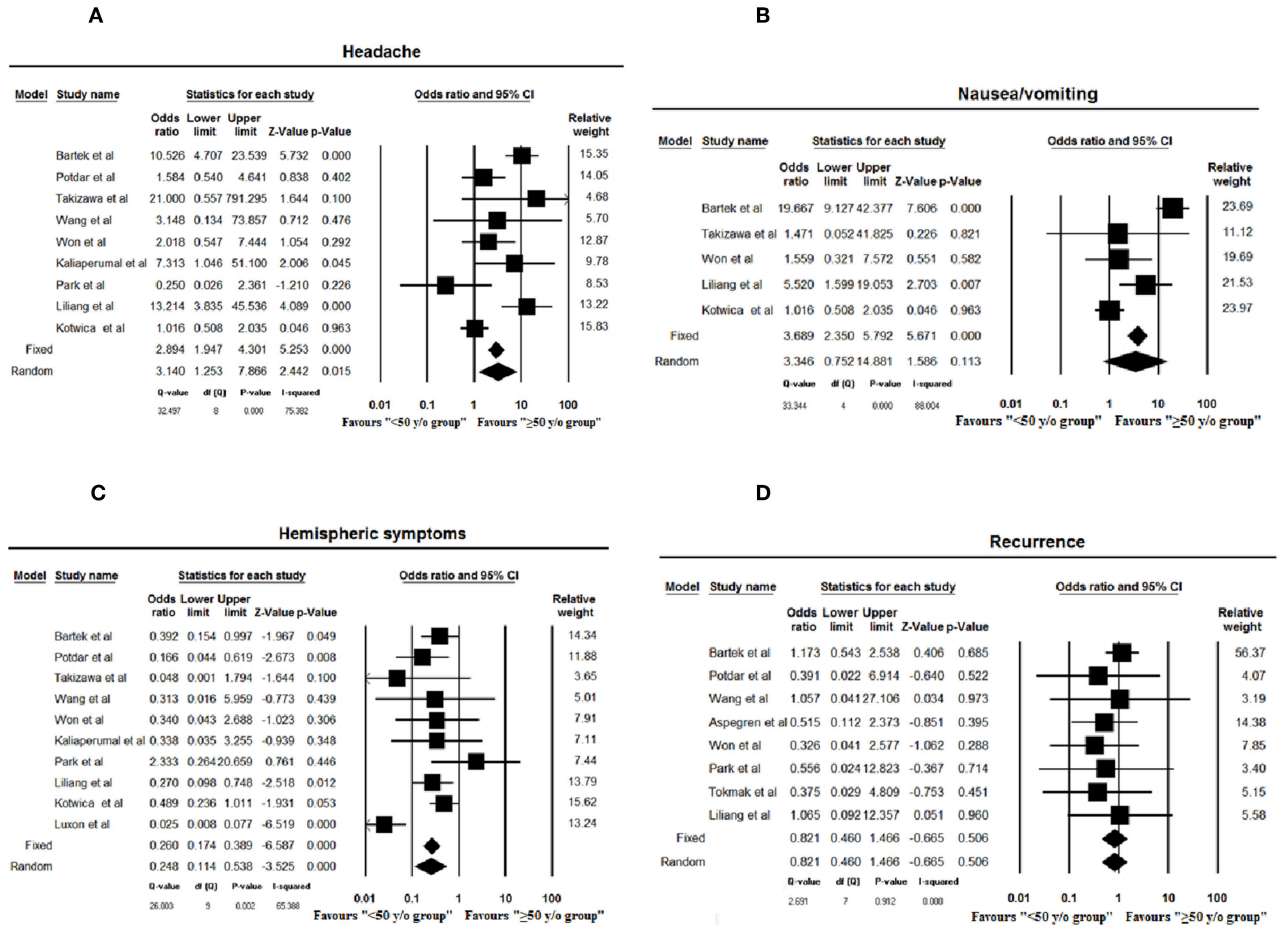
Forest plots of headache **(A)**, nausea and vomiting **(B)**, hemispheric symptoms **(C)**, and recurrence rate **(D)** assessed in meta-analysis (≤50 years as age cut-off). Squares and horizontal bars indicate point estimate and 95% confidence intervals of odds ratio/standardized difference in means in each study, respectively. Diamonds indicate the summary estimates that are calculated as per random effects model.

Fourteen studies including the current study were included in the meta-analysis (4, 18–32). Date of publication ranged from 1979 to 2018. Among a total of 2,440 patients, 282 were classified as younger (<50 y/o). Cohort size, male sex, headache, nausea/vomiting, hemispheric symptoms, papilledema, recurrence, history of trauma, and hematoma thickness are summarized for each group (Supplementary Tables 1, 2). Younger patients are significantly more likely to present with headache (*p* = 0.015) while the hemispheric symptoms are more likely to be experienced by the patients ≥50 y/o (*p* < 0.001, Figure 2).

**Figure 3:**
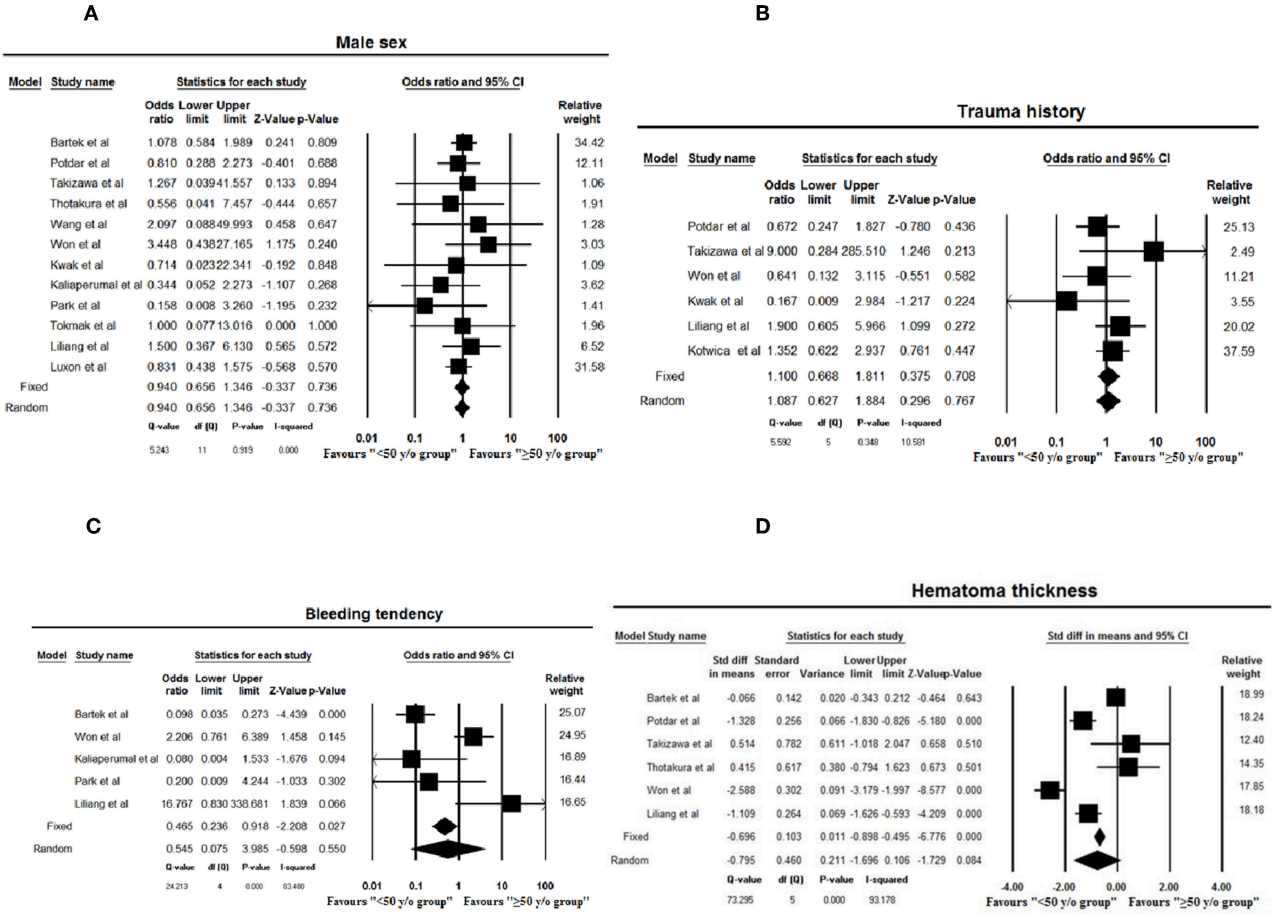
Forest plots of male sex **(A)**, history of trauma **(B)**, bleeding tendency **(C)**, and hematoma thickness **(D)** assessed in meta-analysis (≤50 years as age cut-off). Squares and horizontal bars indicate point estimate and 95% confidence intervals of odds ratio/standardized difference in means in each study, respectively. Diamonds indicate the summary estimates that are calculated as per random effects model.

No statistical differences were found concerning hematoma thickness (*p* = 0.08) or nausea/vomiting (*p* = 0.11), gender (*p* = 0.74), recurrence rate (*p* = 0.51), history of trauma (*p* = 0.77), and bleeding tendency (*p* = 0.55) between age groups (Figures 2, 3). No publication bias was noted among the included studies for any variable as is evident from the gross symmetry of the funnel plots (Supplementary Figures S1, S2).

**Figure 4:**
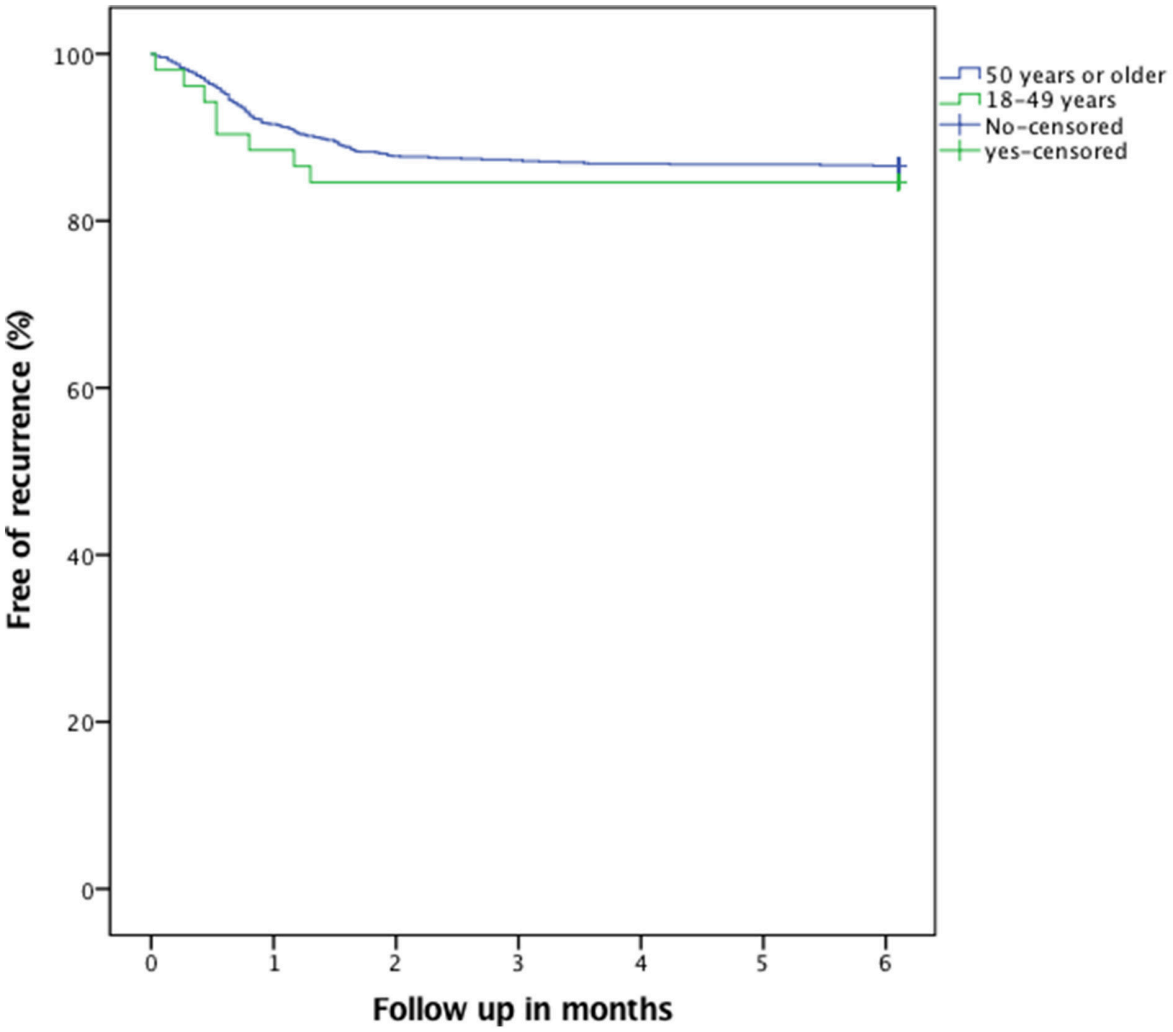
Kaplan-Meier plot illustrating recurrence (*p* = 0.64).

**Figure 5:**
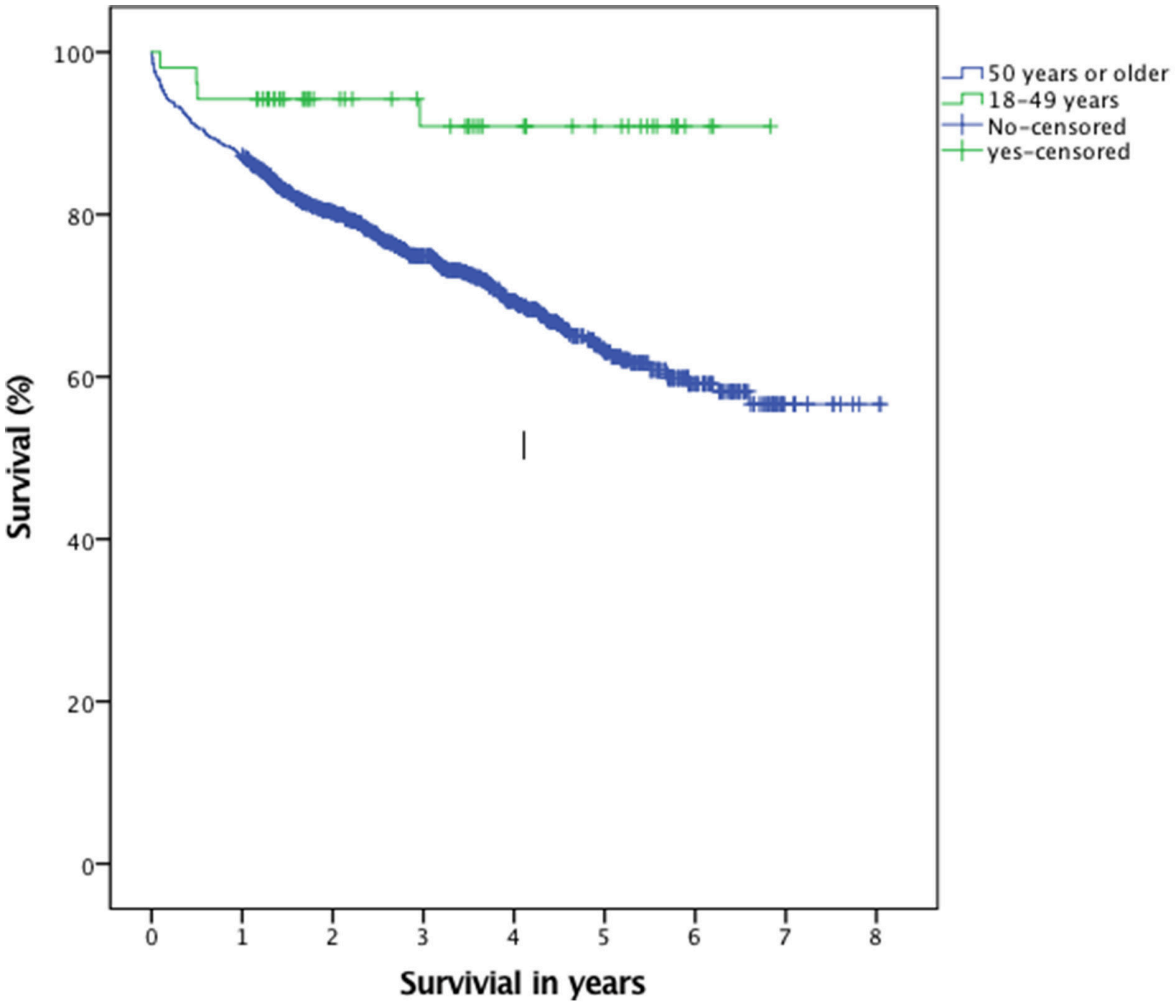
Kaplan-Meier plot illustrating overall survival (*p* = 0.002).

## Discussion

This multicenter, population-based study is to the best of our knowledge the largest cohort study in literature on young patients with cSDH, demonstrating that younger patients (<50 y/o) with cSDH present more often with signs of increased intracranial pressure, while patients ≥50 y/o more often present with neurological deficits. Patients <50 y/o have similar rates of recurrence and perioperative morbidity as compared to patients ≥50 y/o. The diversity in clinical presentation depending on patient age is important for correct and timely diagnosis of cSDH. And although a head scan should be considered in cases of persisting signs of headache, nausea and vomiting without neurological deficits in young adults, one needs to keep in mind the incidence of post-concussion syndrome which is 50% at 1 month and 15% at 1 year when assessing patients (33). Further, to the best of our knowledge, we have conducted the first meta-analysis to compare the clinical pattern in patients <50 y/o vs. ≥50 y/o with cSDH.

### Baseline and Per-Operative Characteristics

The baseline characteristics between groups did not differ significantly except from the younger having less comorbidities, being more independent and using less of both anti-platelets and anticoagulants. Despite increased use of antiplatelet and anticoagulant drugs in the patients ≥50 y/o, the meta-analysis suggests no significant increase in bleeding tendency in this age group.

Between 50 and 80 years of age, it has been shown that the weight of the brain decreases approximately 200 grams (34). Further, the space between the brain and skull increases from 6 to 11% of the total intracranial space (35). As such, this offers greater space for a hematoma to expand without necessarily increasing the intracranial pressure (2, 4). We and others have observed larger on average hematoma diameter in the patients ≥50 y/o, which is logical from a biological point of view. This is even substantiated by the fact that there were 7.7% of younger patients operated with burr hole only (compared to 0.6% of patients ≥50 y/o patients), since the lack of subdural “space” and early brain realignment prevented any drainage being placed. Finally, probably due to the increased risk of adverse events associated to the use of general anesthesia in older patients, local anesthesia + sedation was the preferred method of per-operative sedation (36).

### Clinical Presentation

The clinical presentation was dominated by signs/symptoms of raised intracranial pressure in the patients <50 y/o; headache and vomiting—both which has been previously reported by others as being the cardinal symptoms in young patients with cSDH (1, 2, 4, 5). This is likely explained by a mass effect without significant age-related atrophy, in line with findings that age up to 49 years had no significant influence on the weight of the human brain (10). Thus, symptoms arrive earlier (smaller hematomas) and apparently before neurological deficits occur in most cases, but the midline shift is similar between age groups.

Our findings are consistent with the existing literature as evident from the meta-analysis results, with the younger population more likely to present with headache, and nausea and vomiting, in contrast to the hemispheric symptoms which are more commonly a finding in patients ≥50 y/o.

### Outcome

Similar recurrence rate for cSDH was observed in our study consistent with the meta-analysis results. In the present study, clinical and radiological post-operative controls were less frequently undertaken in the patients ≥50 y/o—which is in line with our belief that there might be a higher threshold to diagnose patients ≥50 y/o, and especially the very old may more often be institutionalized to begin with.

The frequency of adverse events correlated to surgical treatment of cSDH in the younger has been reported to be similar when compared to older or even those extremely old (4, 9), although adverse events are generally seldom reported at all in series concerning the young cSDH patients (2). In our material, although non-significant, patients ≥50 y/o had a higher frequency of overall complications (9.0% vs. 3.8%) than the patients <50 y/o. This becomes even more evident when only looking at serious adverse events (defined as Landriel grade ≥3) which only occurred among the patients ≥50 y/o (*n* = 37, 3.1%).

With respect to mortality, it has been reported in literature that cSDH in the old may reveal and/or exacerbate underlying medical condition leading to poor outcome, a so-called “sentinel health event” as described by Miranda et al. (37), which also demonstrated a lower functional status in older patients at discharge, than when they were first admitted. Nevertheless, we found no significant difference in 30 and 90 days mortality between groups, although a difference in overall survival was noted (*p* = 0.002).

### Strengths and Limitations

Limitations and potential sources of bias associated with retrospective data analysis are present in the current study. Another limitation is the lack of etiological data as well as long-term follow-up data and functional status postoperatively. Nevertheless, our series represents the largest consecutive series of patients <50 y/o operated for cSDH, with a population-based setting making it less at risk of selection bias. Also, our study only takes into account recurrences leading to reoperation—which might have introduced bias in terms of detection and selection.

Further, we have conducted the first meta-analysis comparing clinical profile of patients <50 y/o vs. patients ≥50 y/o with cSDH. The pooled sample size was considerable, with 2,440 patients from 14 studies, which increased the power of our study. No obvious publication bias were detected which added to the reliability of this analysis. Presence of a significant heterogeneity between the included studies is one of the limitations of our study.

## Conclusion

In this multicenter, population-based study of patients with cSDH, supplemented by a meta-analysis based on current literature, we demonstrate a significant difference in clinical presentation of patients <50 y/o vs. patients ≥50 y/o with cSDH. Also, we demonstrate comparable morbidity-, recurrence- and peri-operative mortality rates between groups. It seems reasonable to conclude that attention to clinical presentation is crucial for timely diagnosis of patients with cSDH.

## Ethics Statement

An approval from the Stockholm regional ethical review board in Sweden (EPN 2013/591-31/1) and The Regional Committee for Medical and Health Research Ethics in Central Norway (2011/2050) was obtained prior to study initiation.

## Author Contributions

JB, KS, and AJ designed the study. JB, KS, SD, LS, HK, FS, PF, CC, and AJ performed the data gathering, analysis, and interpretation. JB, KS, and SD drafted the manuscript. JB, KS, SD, LS, HK, FS, and PF critically revised the manuscript under the supervision of CC and AJ.

### Conflict of Interest Statement

The authors declare that the research was conducted in the absence of any commercial or financial relationships that could be construed as a potential conflict of interest.

## Supplementary Material

Supplementary Figure S1 Funnel plots for headache **(A)**, nausea and vomiting **(B)**, hemispheric symptoms **(C)**, and recurrence rate **(D)** demonstrating gross symmetry. Blue circles: observed studies, Red plot: plot for both observed and imputed studies from Duval and Tweedie's trim and fill analysis.

Supplementary Figure S2 Funnel plots for male sex **(A)**, history of trauma **(B)**, bleeding tendency **(C)**, and hematoma thickness **(D)** demonstrating gross symmetry. Blue circles: observed studies, Red plot: plot for both observed and imputed studies from Duval and Tweedie's trim and fill analysis.

Supplementary Table 1 Characteristics of studies with patients with cSDH in the patients <50 y/o age. –, Not reported, ^*^current study.

Supplementary Table 2 Characteristics of studies with patients with cSDH in the patients ≥50 y/o age. –, Not reported, ^*^current study.
