# Supplementary figures and images for: Clinical Course in Chronic Subdural Hematoma Patients Aged 18–49 Compared to Patients 50 Years and Above: A Multicenter Study and Meta-Analysis
Source: Front Neurol. 2019 Apr 5;10:311. doi: 10.3389/fneur.2019.00311 (PMC6459941; doi:10.3389/fneur.2019.00311)

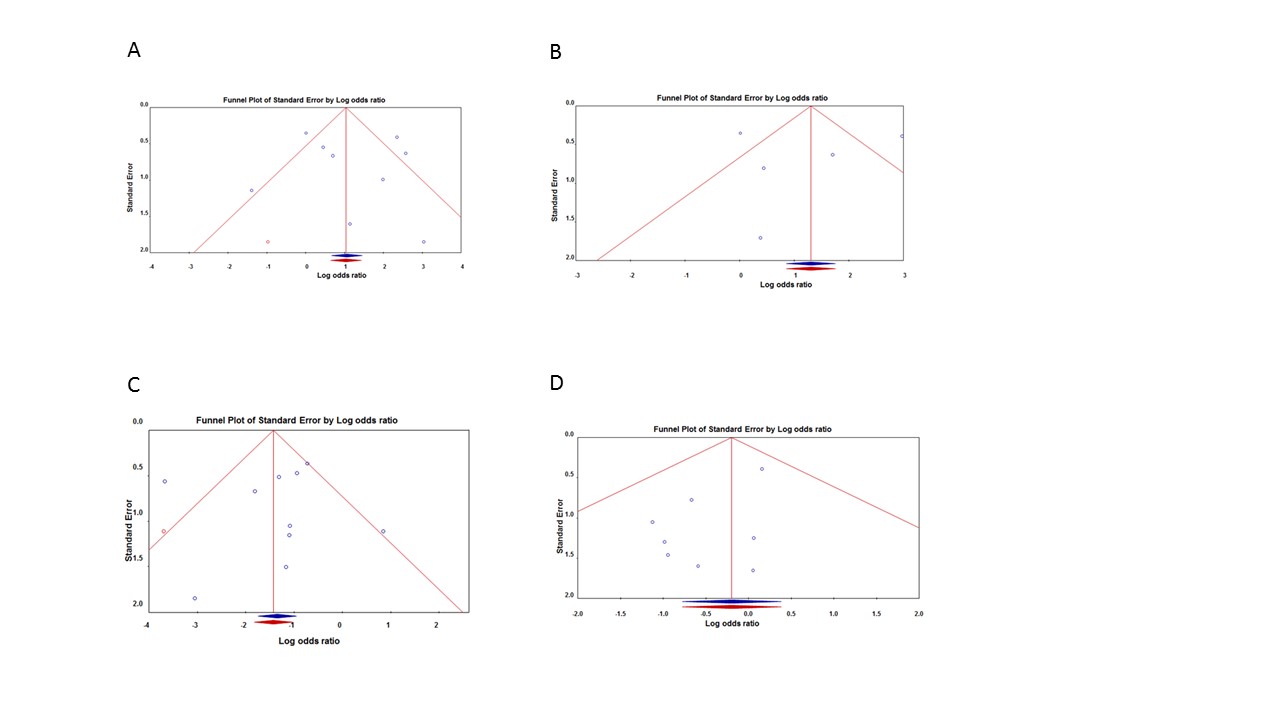

Supplement: Supplementary Figure S1 — Funnel plots for headache (A), nausea and vomiting (B), hemispheric symptoms (C), and recurrence rate (D) demonstrating gross symmetry. Blue circles: observed studies, Red plot: plot for both observed and imputed studies from Duval and Tweedie's trim and fill analysis. [file Image_1.JPEG]

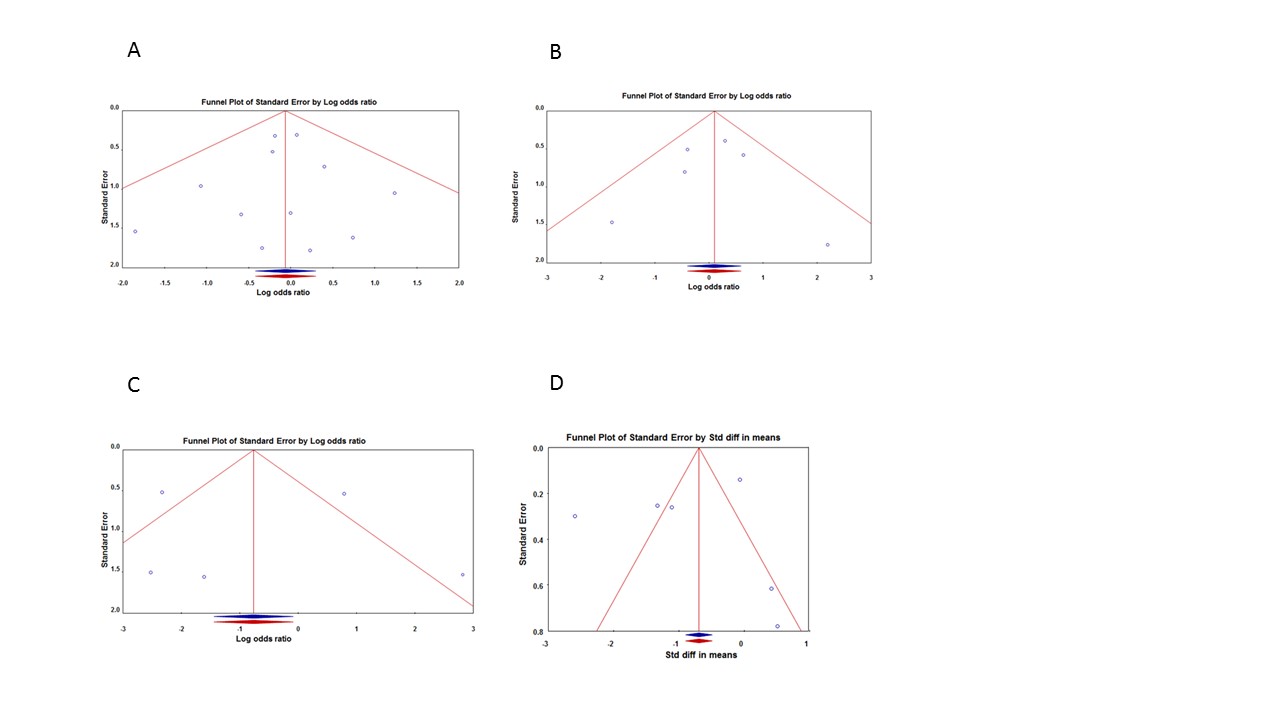

Supplement: Supplementary Figure S2 — Funnel plots for male sex (A), history of trauma (B), bleeding tendency (C), and hematoma thickness (D) demonstrating gross symmetry. Blue circles: observed studies, Red plot: plot for both observed and imputed studies from Duval and Tweedie's trim and fill analysis. [file Image_2.JPEG]

**Supplementary Table 1.**


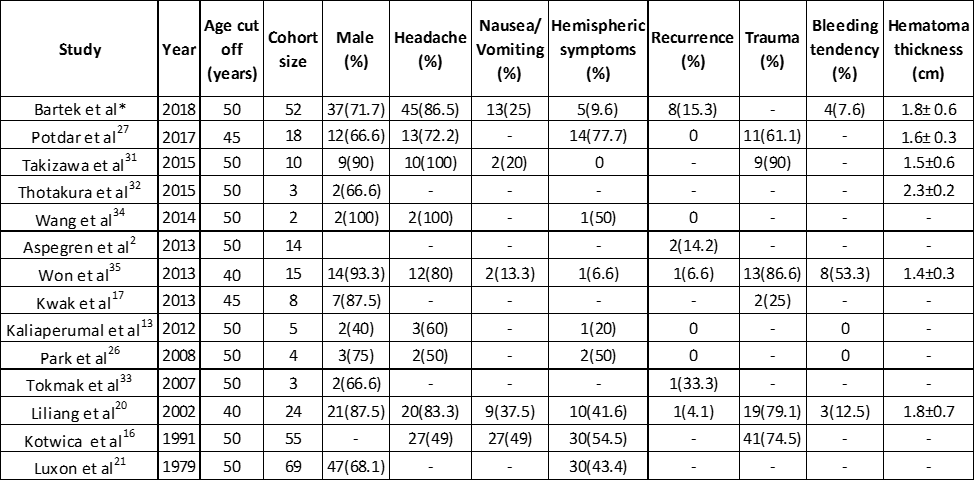


**Supplementary Table 2.**


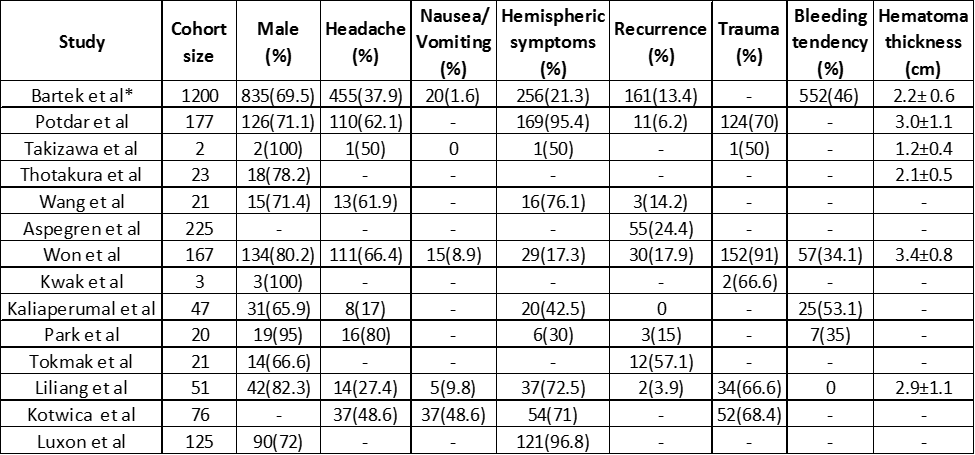

Supplement: Supplementary Table 1 — Characteristics of studies with patients with cSDH in the patients <50 y/o age. –, Not reported, *current study. [file Data_Sheet_1.docx]
